# A Novel Mouse Model of *Schistosoma haematobium* Egg-Induced Immunopathology

**DOI:** 10.1371/journal.ppat.1002605

**Published:** 2012-03-29

**Authors:** Chi-Ling Fu, Justin I. Odegaard, De'Broski R. Herbert, Michael H. Hsieh

**Affiliations:** 1 Department of Urology, Stanford University School of Medicine, Stanford, California, United States of America; 2 Department of Pathology, Stanford University School of Medicine, Stanford, California, United States of America; 3 Division of Immunobiology, Cincinnati Children's Hospital Medical Center, Cincinnati, Ohio, United States of America; NIAID/NIH, United States of America

## Abstract

*Schistosoma haematobium* is the etiologic agent for urogenital schistosomiasis, a major source of morbidity and mortality for more than 112 million people worldwide. Infection with *S. haematobium* results in a variety of immunopathologic sequelae caused by parasite oviposition within the urinary tract, which drives inflammation, hematuria, fibrosis, bladder dysfunction, and increased susceptibility to urothelial carcinoma. While humans readily develop urogenital schistosomiasis, the lack of an experimentally-tractable model has greatly impaired our understanding of the mechanisms that underlie this important disease. We have developed an improved mouse model of *S. haematobium* urinary tract infection that recapitulates several aspects of human urogenital schistosomiasis. Following microinjection of purified *S. haematobium* eggs into the bladder wall, mice consistently develop macrophage-rich granulomata that persist for at least 3 months and pass eggs in their urine. Importantly, egg-injected mice also develop urinary tract fibrosis, bladder dysfunction, and various urothelial changes morphologically reminiscent of human urogenital schistosomiasis. As expected, *S. haematobium* egg-induced immune responses in the immediate microenvironment, draining lymph nodes, and systemic circulation are associated with a Type 2-dominant inflammatory response, characterized by high levels of interleukin-4, eosinophils, and IgE. Taken together, our novel mouse model may help facilitate a better understanding of the unique pathophysiological mechanisms of epithelial dysfunction, tissue fibrosis, and oncogenesis associated with urogenital schistosomiasis.

## Author Summary

Urogenital schistosomiasis (infection with parasitic *Schistosoma haematobium* worms, the most common human-specific *Schistosoma* species globally) affects over 112 million people worldwide. *S. haematobium* worms primarily lay eggs in the bladder, upper urinary and genital tracts, and the host immune response to these eggs is considered to cause almost all associated disease in these organs. Resulting conditions include hematuria (bloody urine), urinary frequency, fibrosis (internal scarring) of the urinary tract, increased risk of bladder cancer, and enhanced susceptibility to contracting HIV. Approximately 150,000 people die annually from *S. haematobium*-induced obstructive kidney failure alone, making this species one of the deadliest worms worldwide. Despite the importance of *S. haematobium*, a lack of an experimentally manipulable model has contributed to the paucity of research focusing on this parasite. We have circumvented the barriers to natural *S. haematobium* oviposition in the mouse bladder by directly microinjecting parasite eggs into the bladder wall. This triggers inflammation, hematuria, urinary frequency, fibrosis, egg shedding, and epithelial changes that are similar to that seen in clinical *S. haematobium* infections. Our model may provide new opportunities to better understand the basic molecular and cellular immunology of urogenital schistosomiasis and thereby contribute to the development of new diagnostics and therapeutics.

## Introduction

Schistosomal infections plague more than 240 million people worldwide. The most prevalent anthropophilic schistosome species globally, *Schistosoma haematobium*, accounts for nearly half of that number, primarily in sub-Saharan Africa and the Middle East [1]. *S. haematobium* infects humans through direct skin penetration by aquatic cercariae that emerge from *Bulinus truncatus*, the intermediate snail host. After entering the human host, the parasite rapidly migrates into the circulation as a schistosomulae, matures, and subsequently lodges in the venous plexus of the bladder where male-female worm pairs mate and produce eggs for years to decades. While in rare cases ectopic *S. haematobium* oviposition causes pathology outside of the urogenital tract, the vast majority of infections result in urogenital schistosomiasis. Although the symptoms are varied, the bulk of the morbidity and mortality of urogenital schistosomiasis can be ultimately attributed to the host immune response against *Schistosoma* eggs deposited within the walls of the urinary tract. This inflammation leads to: 1) compromise of urothelial integrity promoting urinary tract infections [2]–[7], hematuria, and protein-wasting [2]; 2) urothelial changes leading to carcinogenesis [8], [9]; and 3) urinary tract fibrosis causing bladder dysfunction, obstruction, infection, and renal failure [10], [11]. In fact, the annual death toll of 150,000 due to urogenital schistosomiasis-induced obstructive renal failure makes *S. haematobium* one of the most lethal worms worldwide [12].

Despite the global burden of urogenital schistosomiasis, there remains little known about the basic mechanisms underlying the pathophysiology of this disease [13]. This is primarily due to the lack of an experimentally tractable animal model. Indeed, the majority of research in schistosomiasis has focused on *S. mansoni* infections in mice, wherein the entire life cycle can be recapitulated. In contrast, the development of a mouse model of urogenital schistosomiasis, long pursued by investigators in the field, has historically failed due to the inability of *S. haematobium* cercariae to efficiently mature and migrate to the bladder venous plexus in the mouse [14], [15]. Thus, *S. haematobium* research is largely limited to primate [16] and non-murine rodent models [17], [18]. Primate models, while capable of faithful recapitulation of urogenital schistosomiasis, are prohibitively expensive and difficult to manipulate. Extant non-murine rodent models (e.g. hamster), in contrast, develop clinical outcomes which can differ dramatically from the human disease. These models also suffer from a paucity of species-specific tools.

Herein we report the development of a robust, highly manipulable mouse model of urogenital schistosomiasis achieved by the microinjection of viable *S. haematobium* eggs directly into the bladder wall. This model faithfully and reproducibly recapitulates some of the salient features of the human disease including inflammatory cell activation and infiltration, urinary tract granuloma formation and fibrosis, urinary dysfunction, systemic Type 2 immune activation, and egg excretion in urine. To our knowledge, this is the first experimentally tractable mouse model of urogenital schistosomiasis. Moreover, we provide direct evidence that egg deposition alone is sufficient to reproduce several important aspects of urogenital schistosomiasis, even in the absence of the other life stages of this important human pathogen.

## Results

### Intramural *S. haematobium* egg injection induces reproducible granuloma formation and maturation

**Figure 1:**
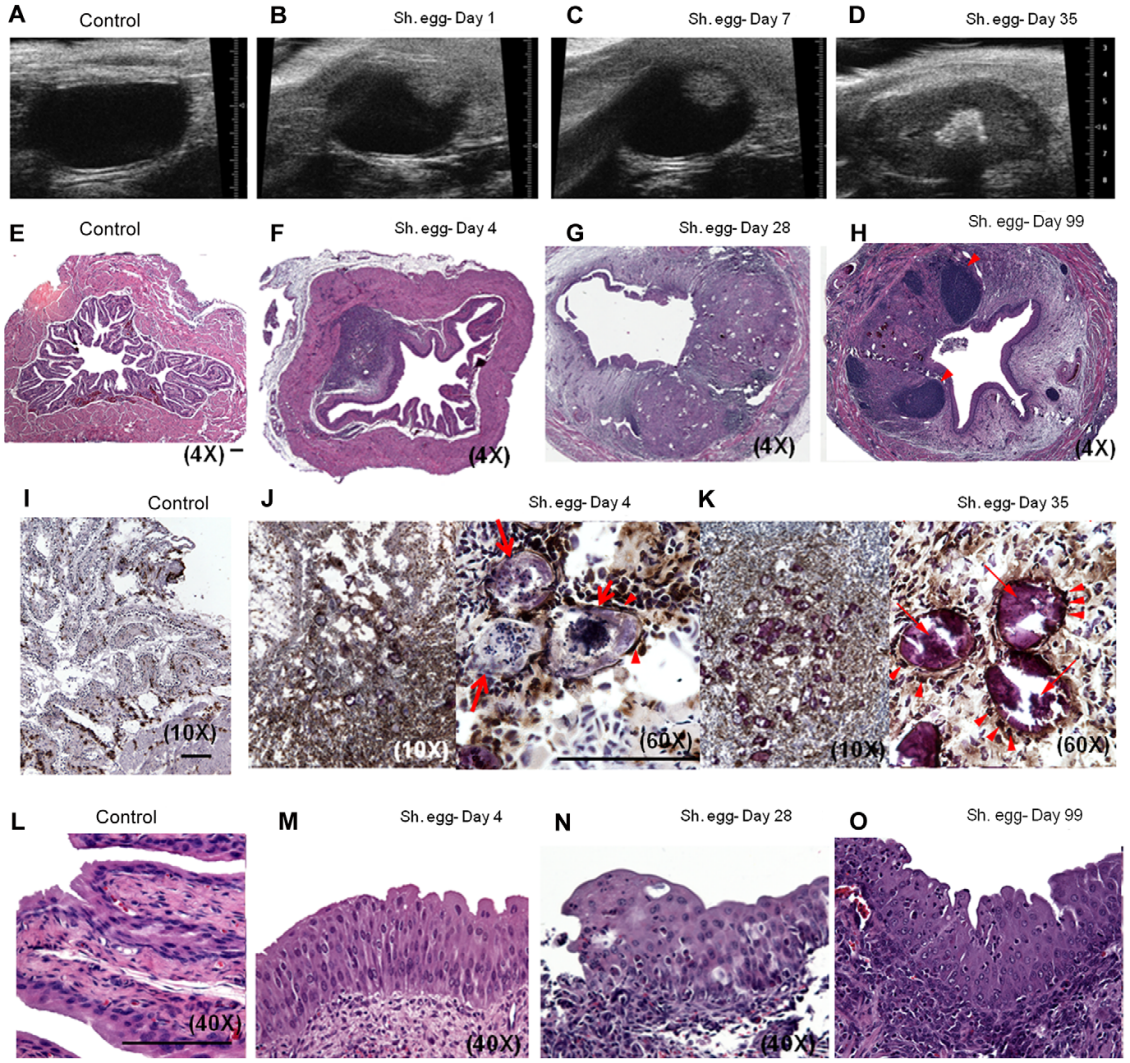
Bladder wall injection of *S. haematobium* eggs results in synchronous granuloma formation. Intramural injection of *S. haematobium* eggs results in rapid and localized injection site response followed by progressive expansion and consolidation over several weeks (serial micro-ultrasonography of a single representative animal, **A–D**; histology, **E–H**). Lymphoid follicles in **H** are marked with arrowheads. Scale bars for panels E, I, J, and L (lower right hand corner for each) are 100 microns long. Immunohistochemical characterization demonstrates central macrophage granuloma formation around injected eggs (arrows) with peripheral accumulation of other inflammatory cells (**I–K**, anti-CD68 brown). Epithelioid cells (activated macrophages) are indicated with arrowheads. Egg injection also induces early and sustained urothelial hyperplasia with reactive nuclear changes (**L–O**).

Although the morbidity associated with chronic *S. haematobium* infection is considered to result from egg deposition into the bladder wall, it is currently unclear whether oviposition alone, in the absence of adult worms, is necessary and sufficient for the bladder pathology associated with urogenital schistosomiasis. To address this issue, we directly microinjected viable *S. haematobium* eggs into the anterior bladder walls of female BALB/c mice. Serial transabdominal micro-ultrasonography paired with histologic verification demonstrated the development of injection site granulomata over time (Figure 1A–H and Video S1). The initial injection site response resolved entirely by day 4 in animals injected with egg-free control vehicle (Figure 1E); whereas egg-injected animals demonstrated a persistent mixed inflammatory infiltrate (Figure 1F) with a hypoechoic appearance on micro-ultrasonography (i.e., low density, grey mural nodules, Figure 1B). Over the following 4 weeks, the egg-associated mixed inflammatory infiltrate expanded and organized into a well-defined, egg-centered granuloma surrounded by peripheral eosinophils and neutrophils and containing distinct lymphoid follicles (Figure 1G). Robust granulomata were still present 99 days after egg injection (Figure 1H). These organized, dense lesions were correspondingly hyperechogenic on micro-ultrasonography (bright nodules, Figure 1D). Granuloma formation was neither sex- nor strain-specific, since male and C57BL/6 and C3H/He mice also developed granulomata after egg injection (unpublished data). The granulomatous character of the egg-associated lesions was confirmed by immunohistochemistry for CD68 (Figure 1I–K), which demonstrated complete encapsulation of the eggs by CD68-positive epithelioid cells (i.e., syncytial macrophages, Figure 1K). Similar to human disease, granuloma development is accompanied by eosinophiluria [19]–[24] and hematuria [25] (Figure S1). Additionally, by post-injection day 4 the urothelium demonstrated pronounced egg-dependent hyperplasia and squamous metaplasia that persisted throughout the experimental time course (Figure 1L–O and data not shown). These changes were present predominantly in the urothelium overlying the egg granuloma (Figure 1F–H), suggesting a highly localized microenvironmental effect. Importantly, these urothelial features closely parallel those observed in urogenital schistosomiasis [26].

The observed pathology was not likely confounded by surgical complications. In more than 100 consecutive injections performed by four independent surgeons, no bladder perforation, extravesical egg deposition, or significant post-injection sequelae were observed. Micro-ultrasonographic and histologic analysis confirmed reproducible egg delivery to the same submucosal tissue plane. Moreover, 20% of mice shed eggs in their urine within one week of egg injection, which recapitulates egg shedding in infected humans (data not shown).

### Intramural *S. haematobium* egg injection induces bladder fibrosis and urinary dysfunction

**Figure 2:**
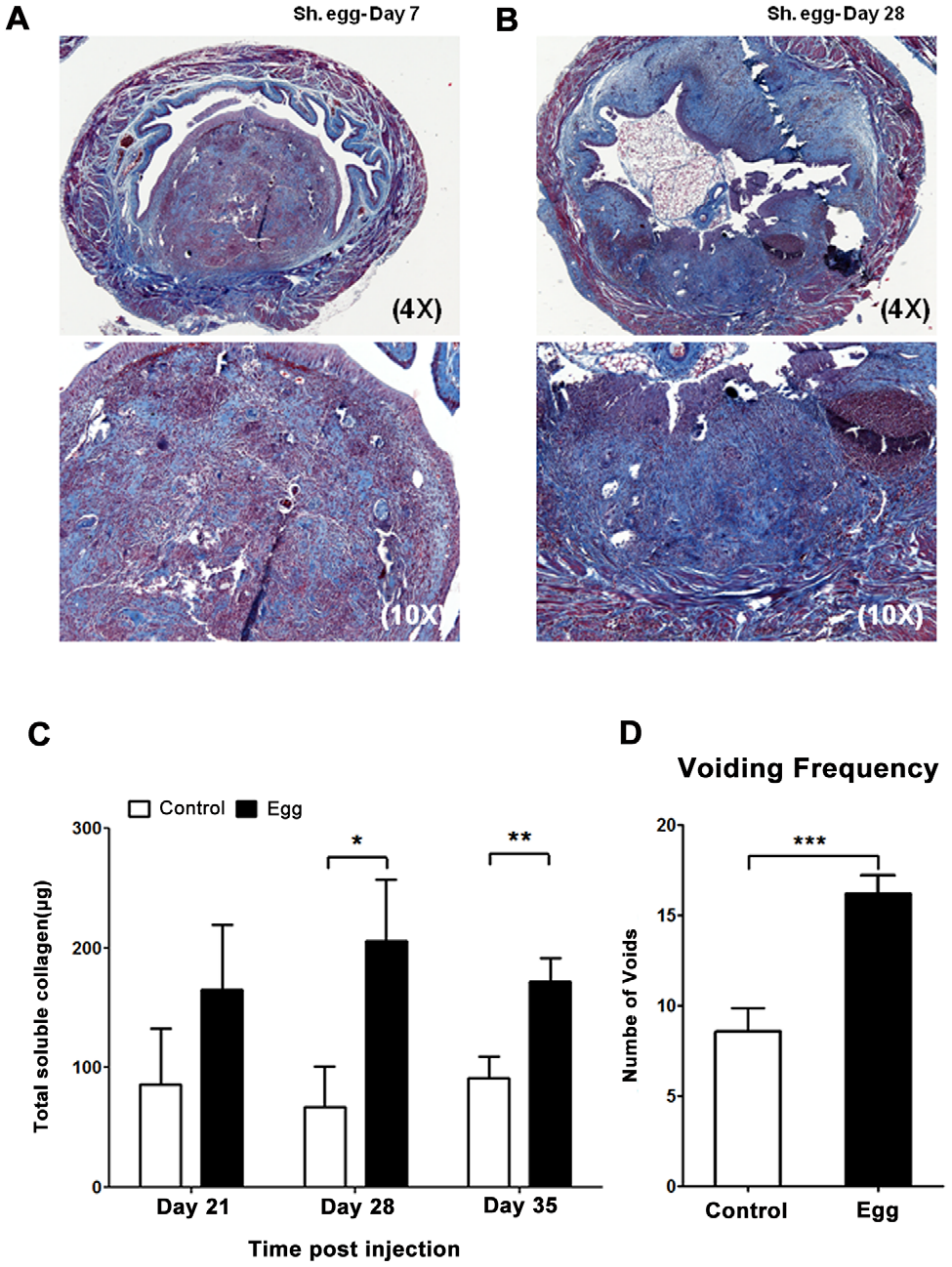
*S. haematobium* egg-injected bladders develop fibrosis and urinary dysfunction. Egg-injected bladders demonstrate histologically-apparent fibrosis within granulomata beginning at post-injection day 7 (**A**, Masson's trichrome stain, collagen stains blue). Later time points demonstrate increased collagen staining area and intensity (**B**). Total bladder soluble collagen content correlates with histologic evaluation (**C**). Egg-injected mice demonstrate increased urinary frequency (1 week post injection, **D**).

The host response to *S. haematobium* eggs in the human urogenital tract involves a fibroproliferative response which is thought to drive ureterovesical obstruction and bladder dysfunction, two major sources of morbidity associated with infection [27]. To determine whether our approach resulted in the development of bladder fibrosis, we evaluated egg-injected bladders through Masson's Trichrome staining and total extractable collagen assays. By day 7, loose immature collagen was observed within nascent granuloma (Figure 2A). At day 28 post-injection, dense mature collagen was found throughout the granuloma with variable extension into the surrounding bladder tissue (Figure 2B). Control vehicle-injected bladders demonstrated little or no collagen staining (data not shown). In addition, total bladder soluble collagen content was markedly increased 3–5 weeks post-egg injection (Figure 2C). Finally, *S. haematobium* egg-injected mice exhibited increased voiding frequency relative to control animals (Figure 2D), which is consistent with reports of urinary frequency observed in parasitized humans [28], [29].

### Egg-injected bladders accumulate an eosinophil- and neutrophil-dominated mixed inflammatory infiltrate

**Figure 3:**
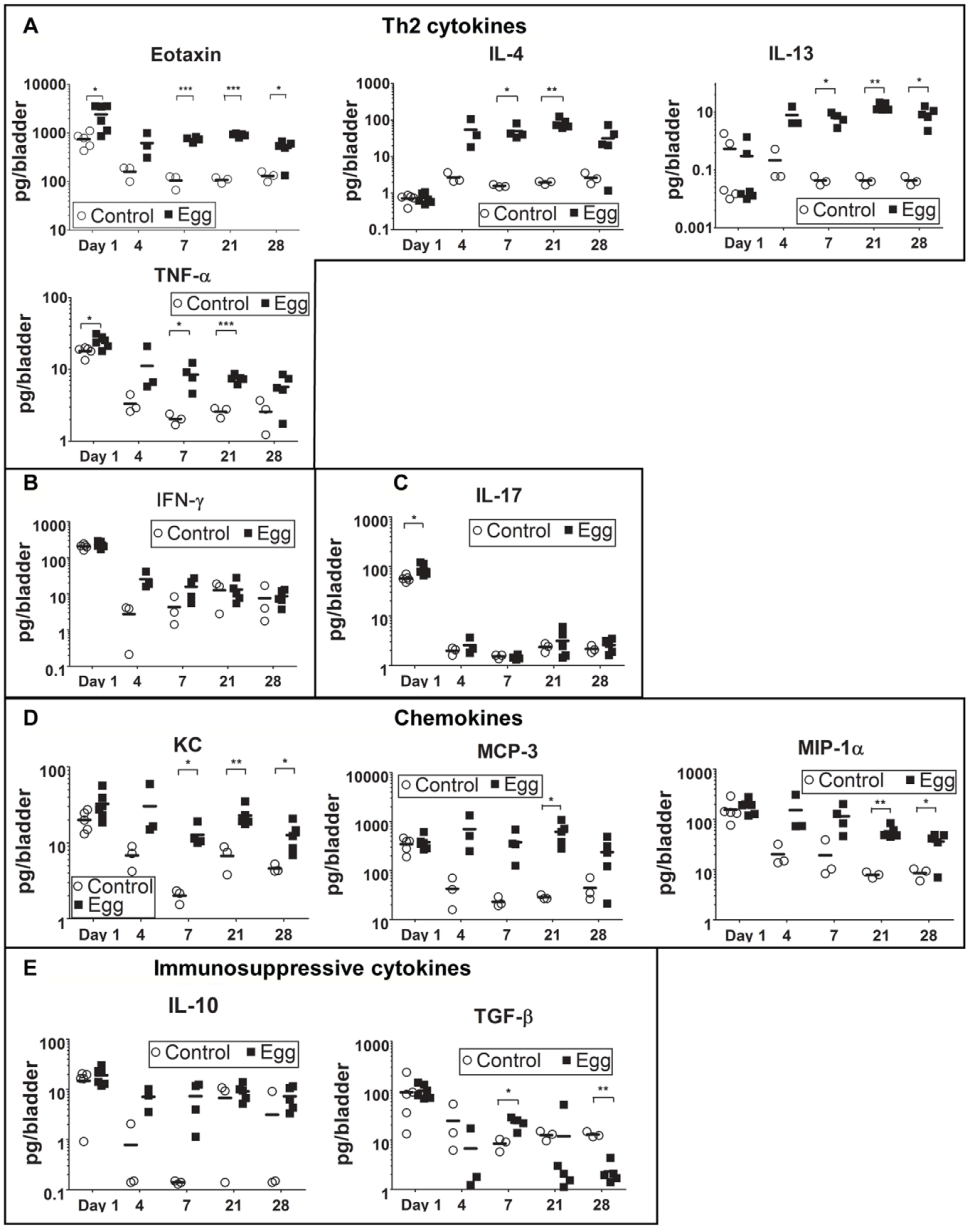
*S. haematobium* egg-injected bladders demonstrate a local Type 2 immune response. Egg-injected bladders feature elevated levels of the Type 2-associated cytokines IL-4, IL-13, and eotaxin (**A**) without significant changes in T_H_1- or T_H_17-associated cytokines (e.g. IFN-γ [**B**] and IL-17 [**C**], respectively). Cytokines and chemokines associated with innate immune activation (e.g. KC, TNF-α, MIP-1α, MCP-3, [**D**]) are persistently elevated relative to control. The immunosuppressive cytokines IL-10 and TGF-β were not clearly differentially regulated in egg- versus control-injected mice (**E**).

The defined and synchronous nature of our egg injection model allowed us to investigate the initial innate immune response to *S. haematobium* egg deposition. Importantly, there was an immediate, egg-independent upregulation of many cytokines in response to the injection itself; however, this rapidly resolved over time (Figures 3 and S2). Given that the foremost histologic hallmark of human parasitic infection is eosinophil infiltration, it was expected that eotaxin, a potent eosinophil chemoattractant, was significantly upregulated relative to control-injected bladders (Figure 3A).

**Figure 4:**
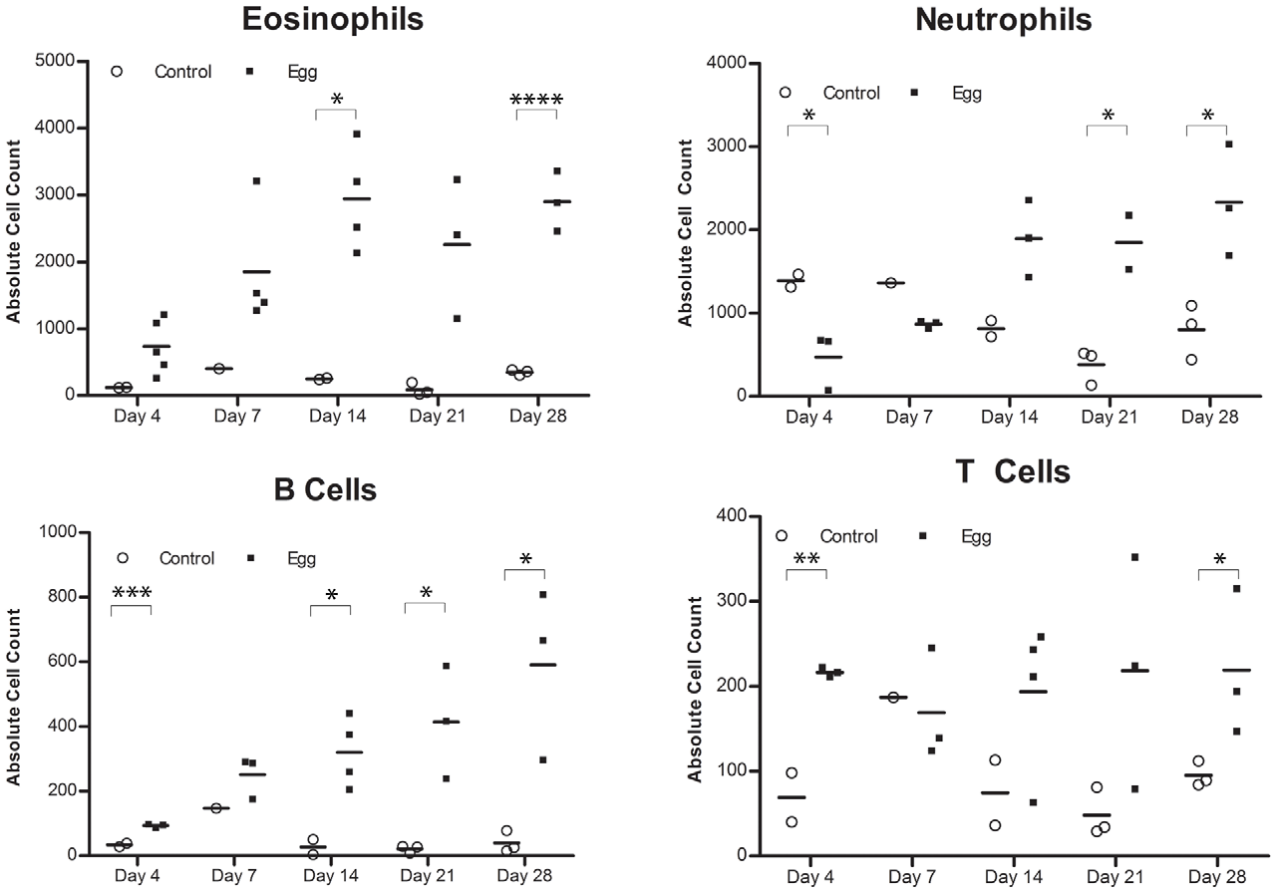
*S. haematobium* egg-injected bladders accumulate a mixed inflammatory infiltrate dominated by eosinophils and neutrophils. Congruent with histologic evaluation, serial flow cytometric analyses of single cell suspensions made from egg-injected bladders demonstrate progressive accumulation of (**A**) eosinophils (SiglecF^+^CCR3^+^), (**B**) neutrophils (CD11b^+^Gr-1^+^), (**C**) B cells (B220^+^), and—to a lesser extent— (**D**) T cells (CD3^+^).

Consistent with the upregulation of eotaxin, we detected a large number of eosinophils (Siglec-F^+^ CCR3^+^) that rapidly infiltrated the injection site and persisted, whereas egg-free control vehicle injections did not produce a significant response (Figure 4A, Figure S1). In addition, there was a marked infiltration of neutrophils (CD11b^+^Gr-1^+^) into the injected bladder wall with kinetics that were similar to eosinophils (Figure 4B). This is consistent with the egg-injected bladder upregulation of neutrophil-associated chemokines such as KC (CXCL1) [30] and MIP-1α (CCL3) [31] (Figure 3). While B-cells (B220^+^) also accumulated at the injection site over time (Figure 4C), we noted a paucity of T cells (CD3^+^), though moderately elevated relative to egg-free controls (Figure 4D). These data are consistent with histologic observations (Figure 1F–H), and suggest that development of egg granulomata in our model features B cell and chemokine-driven innate immune cell infiltration with a relative dearth of T cells.

### Egg-injected bladders demonstrate a distinctly Type 2-biased microenvironment

*S. mansoni* and *S. japonicum* eggs elicit a dominant Type 2 immune response within mouse hosts. Despite the paucity of T cells in our model, we sought to determine whether *S. haematobium* eggs elicited the production of the canonical Type 2 cytokines IL-4 and IL-13. The Luminex multiplexed liquid microbead platform was used to assay total cytokine expression within egg-injected bladders at early time points that corresponded to the initial immune response and nascent granuloma development (Figure 3 and Figure S2). IL-4 and IL-13 were upregulated by day 4 and remained elevated throughout all time points examined (Figure 3A). IL-5 levels were also markedly increased, which is consistent with the role of this Type 2-associated cytokine in eosinophil differentiation, activation, and recruitment [32] (Figure S2). In contrast, T_H_1- and T_H_17-associated cytokines such as IFN-γ and IL-17 remained unaffected by the egg-induced inflammatory response (Figure 3B–C). Consistent with the marked neutrophil and macrophage infiltration of the egg-injected bladder wall (Figures 1 and 3), the innate immunity-associated cytokines TNF-α, KC, MCP-3, and MIP-1α demonstrated early and sustained increases relative to controls (Figure 3A and 3D). Interestingly, egg injection had no effect on IL-10 and TGF-β—immunosuppressive cytokines associated with regulation of tissue fibrosis in other diseases (Figure 3E).

### *S. haematobium* egg injection induces Type 2 responses in draining lymph nodes

**Figure 5:**
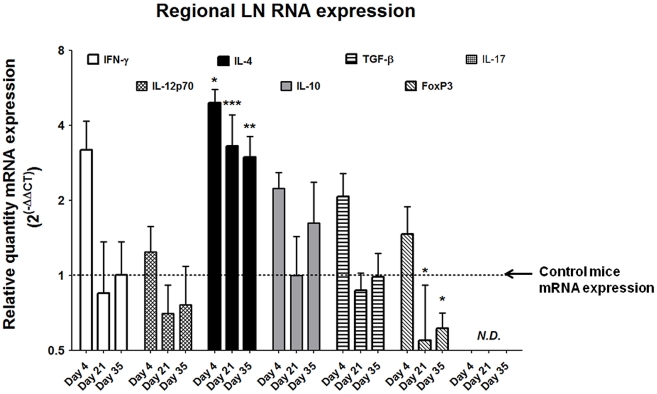
*S. haematobium* egg-injected mice demonstrate Type 2 immune responses in draining lymph nodes. Pelvic lymph nodes from egg-injected mice demonstrate persistently elevated expression of Type 2-associated cytokines (e.g. IL-4) without corresponding increases in the T_H_1-associated cytokines IFN-γ and IL-12. The T_Reg_-associated marker FoxP3 is suppressed late in the experimental time course, while the immunosuppressive cytokines IL-10 and TGF-β remain unchanged. IL-17 was not detected (N.D.).

Despite the relative paucity of T cells within the egg-injected bladder (Figure 4D), the strikingly Type 2 cytokine-biased microenvironment (Figure 3) supported a hypothesis that T helper 2 cells were likely to be involved in the immune response to *S. haematobium* eggs. Indeed, pelvic lymph nodes draining egg-injected bladders demonstrated marked upregulation of the T_H_2-associated cytokine IL-4 throughout the experimental time course, while expression of the T_H_1-associated cytokines IFN-γ and IL-12 was moderately altered due to the injection procedure (Figure 5). Interestingly, at later time points the T_reg_-associated marker FoxP3 was markedly suppressed relative to controls while expression of the immunosuppressive cytokines IL-10 and TGF-β was unchanged. Expression of IL-17 was not detected.

### Egg-injected mice display systemic Type 2-skewed cytokine responses and IgE production

**Figure 6:**
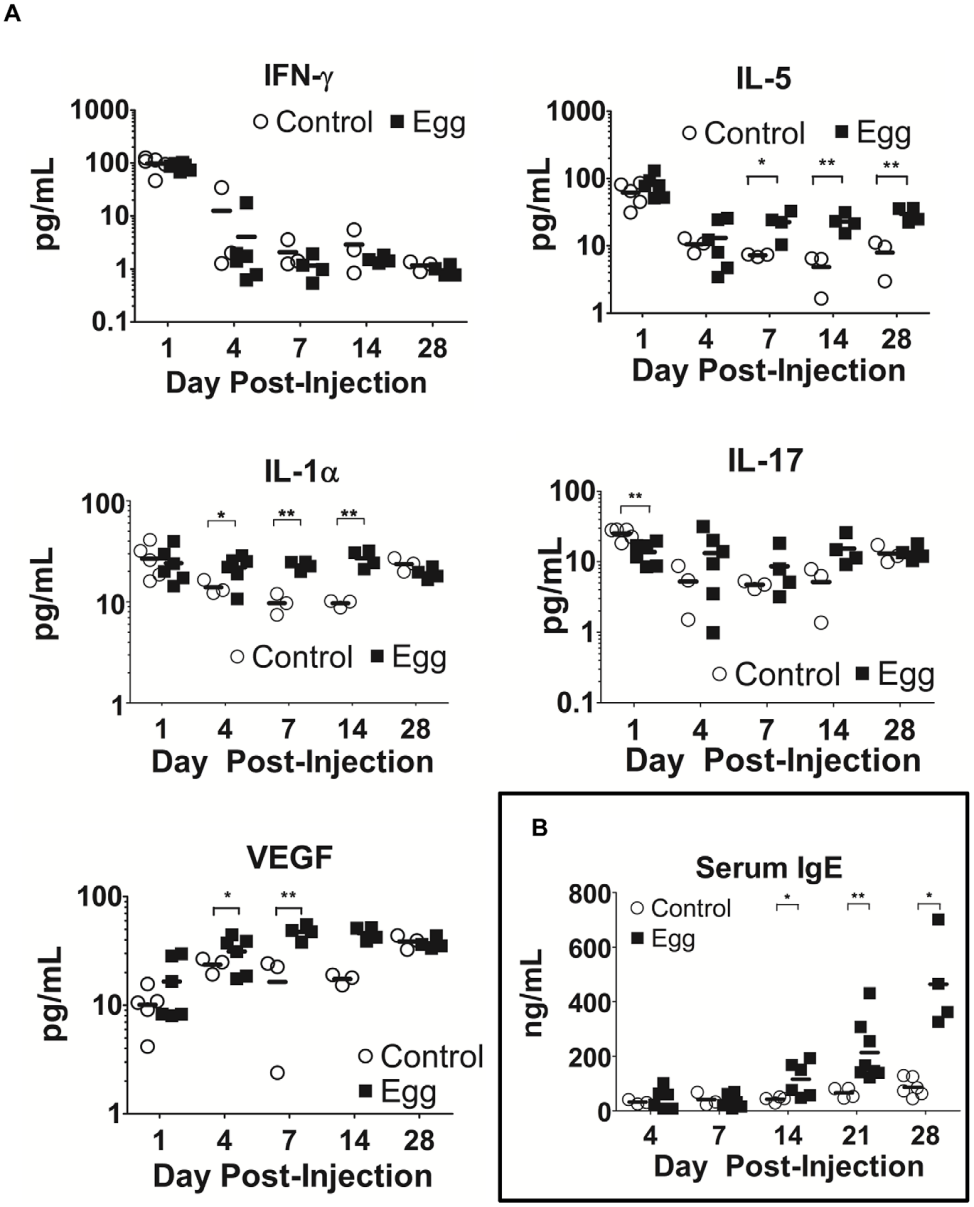
*S. haematobium* egg-injected mice display systemic Type 2 cytokine and immunoglobulin responses. Egg-injected mice demonstrate elevated serum levels of Type 2-associated cytokines (e.g. IL-5) with decreased or unchanged serum levels of T_H_1- and T_H_17-associated cytokines (e.g. IFN-γ and IL-17, respectively, [**A**]). The Type 2 bias in systemic cytokine expression parallels increased IgE production (**B**).

In our model, egg-injected mice demonstrated a reproducible, systemic Type 2-biased immune response similar to that observed in human infection. Serial multiplex serum cytokine profiling demonstrated persistently elevated levels of the Type 2-associated cytokine IL-5, while the T_H_1- and T_H_17-associated cytokines IFN-γ and IL-17, respectively, evinced no such increase (Figure 6A). Congruent with chronic inflammation, the innate immunity-associated cytokine IL-1α was also persistently elevated (Figure 6A). Interestingly, serum levels of VEGF were increased in egg-injected mice, which may have promoted aberrant vasculogenesis and hematuria analogous to human disease [33]. Finally, serum levels of IgE, a quintessential Type 2-associated antibody isotype, were increased beginning at 14 days post egg injection relative to controls, and remain elevated through day 28 post-injection (Figure 6B).

## Discussion

*S. haematobium* infection, i.e. urogenital schistosomiasis, lacks a reliable mouse model despite being the most prevalent form of schistosomiasis and one of the deadliest worm infections worldwide. To address the lack of experimentally amenable tools for investigation of this medically important pathogen, we have developed an improved mouse model of urogenital schistosomiasis. Microinjection of viable *S. haematobium* eggs into the submucosa of the bladder wall elicits pathology similar to certain aspects of the human disease, including inflammatory cell infiltration, granuloma formation, urinary tract fibrosis and dysfunction, and systemic Type 2 immune activation. The focal deposition of eggs and resulting composite granulomata observed in this model recapitulates certain aspects of the immunopathology observed in human disease [34]–[36]. The persistent granulomatous inflammation in our model (at least 99 days after egg injection) parallels the chronicity of human infection. Moreover, the microinjection method features several advantages, including the induction of anatomically precise, synchronous, and reproducible pathology. Although microinjection of the bladder wall of a 20 gram mouse may appear daunting, proper magnification, clean egg preparations, sharp injection needles, and careful surgical technique render it feasible [37]. This is in contrast to existing mouse models of percutaneous or intravenous *S. haematobium* infection, which are prone to ectopic oviposition, variant kinetics, and unreliable disease burden [14], [15].

Current, non-mouse animal models for urogenital schistosomiasis mostly rely on non-human primates [16] and hamsters [17], [18]. Non-human primate models, while capable of high fidelity recapitulation of human disease, are costly and difficult to use. In hamsters transdermally infected with *S. haematobium* cercariae, schistosomula reach the lung by 3 days post-infection, followed by pairing of worms at approximately day 28–29. Oviposition in hamster tissues, primarily lung, liver, intestine, spleen, kidney, and uterus, begins to occur between weeks 7–11 [38]–[42]. Clustered egg deposition (often >20 eggs) results in giant, composite granulomata in the hamster liver. In comparison, *S. mansoni* infections of hamsters result in single egg-based liver granulomata containing more eosinophils, fewer polymorphonuclear leukocytes and histiocytes [43], [44]. Rates of hamster bladder involvement after exposure to *S. haematobium* cercaria are low and inconsistent, ranging from 0 to approximately 60%. Even when bladder oviposition occurs, egg burdens tend to be much lighter than that found in other organs, and less than two-thirds of hamsters with bladder eggs feature urothelial hyperplasia or squamous metaplasia [45]. Thus, hamsters develop clinical pathology which can differ dramatically from human disease, restricting their biological relevance. Hamsters also feature fewer species-specific reagents than mice. Hence, our improved mouse model of urogenital schistosomiasis may prove to be a useful alternative to existing animal models of this disease.

In the course of characterizing our model we noted that significant Type 2 inflammation occurs after egg injection, but this is not accompanied by large numbers of granuloma-associated T cells. Instead, we noted a rapid and pronounced chemokine response ensued following egg inoculation. The rapidity of the eotaxin response may indicate that eosinophils were recruited in the absence of adaptive immunity, perhaps by the secretion of eotaxin from urothelial, endothelial, smooth muscle, or other resident cells that were likely in direct contact with the inoculum. Certainly, urothelial cells can serve as a rapid source of chemokines and other cytokines in response to exposure to microbial antigens [46]. While the precise mechanism by which eosinophils are initially recruited to the sites of *Schistosoma* infection is not known, their later recruitment and accumulation is driven by a local, robust Type 2-biased immune response [47]. Indeed many of the aspects of parasitic morbidity, including those associated with urogenital schistosomiasis, are driven by this immune program. In humans, the parasite microenvironment has been well-characterized in its later, chronic stages; however, the early development and etiologic determinants of this immunologic milieu are poorly understood, most especially in urogenital schistosomiasis. The few T cells present in our model's bladder granulomata may be amplifying and organizing the local immune response, given the development of lymphoid follicles late after egg injection (Figure 1H). Despite the paucity of bladder-infiltrating T cells, the observed increase in IL-4 gene expression in draining lymph nodes (Figure 5) argues in favor of regional activation of T_H_2 cells. Alternatively, it is possible that basophils stimulated by *S. haematobium* egg-derived IL-4 inducing principle from *S. mansoni* eggs [48] (IPSE, originally known as *S. mansoni* chemokine binding protein [smCKBP] [49]) subsequently transit through lymph nodes and secrete IL-4 in these sites [50]. Other potential sources of IL-4 include mast cells and natural killer T (NKT) cells, both of which have been reported to localize to the lymph nodes [51], [52]. The latter cellular subset has been specifically implicated in anti-schistosomal immune responses [53]. Regardless of the source of IL-4, IgE titers increased beginning two weeks after egg injection (Figure 6B), providing consistent evidence for IL-4-dependent B cell isotype switching [54].

Another important observation was the lack of differential regulation of Th1 and Th17 cytokines (Figure 3). Immunologic aspects of natural *S. mansoni* infections of mice feature early Th0 or Th1 responses [55]–[57], with certain inbred mouse strains also exhibiting a propensity for Th17-associated activity [58]. We speculate that the differences between our model and this body of work are due in part to the synchronous granuloma nature of our approach, which does not include the cercarial-, schistosomular-, and worm-triggered immune response. It is also possible that the bladder immune microenvironment differs from other sites of schistosomal infection, namely the lung, liver, and intestinal tract. Further refinements to our model, and combination of our model with natural infection models, will be necessary to dissect out these important questions.

Besides differential cytokine expression, the systemic upregulation of the growth factor VEGF in response to egg injection was particularly striking (Figure 6A). Hematuria is a hallmark of urogenital schistosomiasis, and by definition results when bladder blood vessels and the urothelium break open and communicate with the bladder lumen. We theorize that VEGF triggers disorganized vasculogenesis and results in friable, easily disrupted bladder neovasculature. Interestingly, cervicovaginal lesions associated with urogenital schistosomiasis exhibit increased amounts of sprouting blood vessels and granulation tissue, indicating a possible role for VEGF and/or other vasculogenic influences [59].

The successful mimicry of several pathophysiologic facets of urogenital schistosomiasis by direct egg microinjection suggests that egg deposition alone may be sufficient to recapitulate some of the salient aspects of human disease. We have preliminary evidence that soluble *S. haematobium* egg antigens alone are also capable of generating bladder inflammation (manuscript in preparation). Use of genetically modified eggs in our model will further define the molecular basis of bladder immunopathology [60]. The observed long-term inflammation does not appear to be caused by bacterial or endotoxin contamination of injection solutions, since: 1) solutions are sterile; 2) endotoxin levels are <0.06 EU/dose; and 3) injection of eggs or control vehicle does not result in more TNF production than injection with low endotoxin saline (data not shown).

The availability of an improved animal model of urogenital schistosomiasis is of importance to multiple avenues of study. Firstly, the ability to monitor and manipulate the disease in a host (*Mus musculus*) for which numerous species-specific tools are available enables experimental approaches which were previously inaccessible. Secondly, the reliable reproduction of systemic and urogenital stigmata in our model may allow identification and evaluation of novel diagnostic and therapeutic strategies. Our use of micro-ultrasound is, to our knowledge, the first reported application of this technology for *in vivo* imaging of experimental schistosomiasis. We have successfully employed mouse-specific mass spectrometry and microarray analyses to profile *S. haematobium*-induced host protein and gene expression signatures, respectively (manuscripts in preparation). Egg-specific biomarker studies are ongoing. These approaches have only been possible through use of a mouse model featuring precisely controlled and reproducible *S. haematobium* egg-induced immunopathology. Our model may alleviate the bottleneck on urogenital schistosomiasis research imposed by the scarcity and heterogeneity of infected human samples, particularly bladder and lymphoid tissue.

When combined with other routes of egg injection or transdermal infection with cercariae, our model of schistosome egg-induced, Type 2-associated fibrosis is capable of synchronous fibrosis in multiple anatomic sites including the bladder, liver, and subcutis within the same animal (unpublished data). Cheever et al. have demonstrated that sensitization of mice by adult *S. mansoni* worm antigens enhances the egg-specific, lung immune response [61]. Our model is amenable to testing analogous questions using *S. haematobium* and the bladder. Through this strategy fibrosis in different organ systems may be compared to identify shared and organ-specific mechanisms and potential therapeutic targets. Prior work by others has definitively demonstrated that schistosome egg-induced lung, liver, and intestinal granuloma development is greatly schistosome species-dependent, with differences among *S. haematobium, S. japonicum, S. mekongi*, and *S. mansoni* [62], [63]. In addition, others have reported that hepatic- and lung-associated, *S. mansoni* egg granulomata develop in a highly organ-specific fashion [64]. *S. japonicum* granulomata also evolve in a tissue-specific manner in the liver, lung, and intestinal tract [65]. These reports highlight the critical need to develop *in vivo* models which properly match schistosome species with their tropism for specific host organs.

Additionally, our model of urogenital schistosomiasis presents a unique opportunity to study schistosomiasis-associated carcinogenesis [3], [9], and potentially inflammatory carcinogenesis in general (reviewed by Kuraishy et al. [66]). Urothelial carcinoma associated with *S. haematobium* infection arises in a Type 2-biased inflammatory environment. Approaches combining egg-induced Type 2 immunopathology and inducible models of bladder carcinogenesis represent new methods to study the role of inflammatory bias in carcinogenesis.

Like all experimental models, our approach has limitations. The injection procedure itself induces non-specific upregulation of a number of cytokines at day 1 post-injection (Figures 4, 6, S2, and S3). Use of uninfected hamster tissue homogenates as a control “vehicle” injection only partially mitigates this confounding issue. Accordingly, the cytokine expression observed at day 1 post-injection is likely the result of both parasite-specific and non-specific stimuli. While local immune responses to eggs are directly responsible for much of the observed pathology in *S. haematobium* infection, this activity is part of a larger, systemic immune response elicited in response to multiple life stages of the schistosome. In human disease, *S. haematobium* infection proceeds through cercarial skin invasion, systemic schistosomular circulation and maturation, adult worm mating within the bladder venous plexus, and egg deposition/excretion [67]. This complex natural history provides potential exposure to a broad range of antigens; however, the relative brevity of cercarial persistence and schistosomular circulation (hours to days [68]) and the relative lack of antigenicity of adult worms [69] may limit their contribution to long-term immunopathology. The unexcreted schistosome egg, in contrast, may persist for many years in host tissues and is a well-established, potent immunogen (e.g., soluble egg antigen or SEA [69]). Indeed, the systemic and granulomatous immune response to urogenital schistosomiasis is primarily driven by egg-associated antigens [70], [71]. Our model has methodologic similarities to synchronous granuloma formation induced by bolus injection of eggs into the mouse tail, cecal, or portal vein [64], [72], [73]. Like other synchronous granuloma models, our approach by definition is unsuitable for the study of the cercarial, schistosomular, and worm stages of *S. haematobium*.

In summary, we report the development of an improved mouse model of urogenital schistosomiasis. The ease and robustness of this model make it attractive for potential application to the elucidation of disease mechanisms, discovery of novel diagnostic biomarkers, and evaluation of candidate therapeutics. Additionally, this model is a prospective platform for the study of basic mechanisms of disease such as epithelial dysfunction, fibrosis, and inflammatory carcinogenesis.

## Materials and Methods

### Ethics statement

All animal work has been conducted according to relevant U.S. and international guidelines. Specifically, all experimental procedures were carried out in accordance with the Administrative Panel on Laboratory Animal Care (APLAC) protocol and the institutional guidelines set by the Veterinary Service Center at Stanford University (Animal Welfare Assurance A3213-01 and USDA License 93-4R-00). Stanford APLAC and institutional guidelines are in compliance with the U.S. Public Health Service Policy on Humane Care and Use of Laboratory Animals. The Stanford APLAC approved the animal protocol associated with the work described in this publication.

### Mice

7 to 8 week-old female BALB/c mice were purchased from Jackson Laboratories. All experimental procedures were carried out in accordance with the APLAC protocol and the institutional guidelines set by the Veterinary Service Center at Stanford University.

### *S. haematobium* egg isolation

*S. haematobium*-infected LVG hamsters were obtained from the National Institute of Allergy and Infectious Diseases Schistosomiasis Resource Center of the National Institutes of Health. The hamsters were sacrificed at the point of maximal liver and intestinal *Schistosoma* egg levels (18 weeks post-infection [74]), at which time livers and intestines were minced, homogenized in a Waring blender, resuspended in 1.2% NaCl containing antibiotic-antimycotic solution (100 units Penicillin, 100 µg/mL Streptomycin and 0.25 µg/mL Amphotericin B, Sigma-Aldrich), passed through a series of stainless steel sieves with sequentially decreasing pore sizes (450 µm, 180 µm, and 100 µm), and finally retained on a 45 µm sieve. Control injections were performed using similarly prepared liver and intestine lysates from age-matched, uninfected LVG hamsters (Charles River Laboratories).

### *S. haematobium* egg injection

7 to 8 week-old female BALB/c mice were anesthetized with isoflurane, a midline lower abdominal incision was made, and the bladder exteriorized. Freshly prepared *S. haematobium* eggs (3,000 eggs in 50 µl of phosphate-buffered saline, experimental group) or uninfected hamster liver and intestinal extract (in 50 µl of phosphate-buffered saline, control group) was injected submucosally into the anterior aspect of the bladder dome [37]. Abdominal incisions were then closed with 4-0 Vicryl suture, and the surgical site was treated once with topical antibiotic ointment.

### Micro-ultrasonography

At various time points after bladder wall injection, mice were anesthetized using vaporized isoflurane and their abdominal walls were depilated. Transabdominal images of the bladder were then obtained using a VisualSonics Vevo 770 high-resolution ultrasound micro-imaging system with an RMV 704 scanhead [40 MHz] (Small Animal Imaging Facility, Stanford Center for Innovation in In-Vivo Imaging).

### Bladder histopathologic analysis and collagen measurement

Mice were sacrificed at serial time points 4 to 99 days after bladder wall injection, and bladders processed for routine histology. Morphologic and morphometric analyses were conducted on H&E- and Masson's Trichrome-stained sections. Total collagen content was determined from fresh-frozen (−70°C) bladder homogenates using the Sircol Soluble Collagen Assay Kit (Biocolor, Carrickfergus, United Kingdom) according to the manufacturer's instructions. Collagen concentrations were determined using standard curve analysis. Statistical comparisons were conducted using Student's t-test.

### IgE ELISA

ELISA measurement of serum IgE was performed using manufacturer's instructions (Bethyl Laboratories Mouse IgE ELISA Quantitation Kit). In brief, coating antibody was aliquoted into and allowed to bind to microtiter plate wells. Excess antibody was washed away. Next, blocking solution was added to the wells, allowed to bind, and excess was washed away. IgE standards and experimental samples were added to wells, incubated for an hour at ambient temperature, and washed. HRP detection antibody was added to each well, incubated, and washed. TMB substrate solution was then added to each well, developed for 15 minutes at ambient temperature, and the reactions stopped using Stop Solution. Absorbance of each well was then read on a plate reader at 450 nm.

### Macrophage-specific immunohistochemistry

Five µm sections from OCT-embedded frozen bladders were fixed in 10% buffered formalin phosphate, blocked with 10% horse serum, and incubated overnight at 4°C with a mouse-specific anti-CD68 antibody (BioLegend, San Diego, CA). Sections were then processed and developed using an anti-rat IgG staining kit (Biocare Medical, Concord, CA) according to the manufacturer's instructions and counterstained with hematoxylin.

### Analysis of bladder-associated leukocytes

Freshly-excised bladders from egg-injected mice were minced and incubated with agitation in 0.5% heat-inactivated FBS (Thermo Scientific Hyclone, IL), 20 mM HEPES pH 7, 0.057 Kunitz U/ml DNase I (Sigma-Aldrich), and 1 mg/ml collagenase B (Roche) in RPMI 1640 medium for 1 hr at 37°C [75]. The tissue was then passed through a 70 µm nylon cell strainer to remove undigested tissue and macrocellular debris. After erythrocyte lysis (8.02 mg/ml NH_4_Cl, 0.84 mg/ml NaHCO_3_, and 0.37 mg/ml EDTA in distilled water), 10^6^ cells were treated with mouse anti-CD16/CD32 (clone 2.4G2, BioLegend, San Diego, CA) for 10 min and stained with mouse anti–CD3-PE/Cy7 (clone 145-2C11, BioLegend), anti–CD45 (B220)-FITC (clone RA3-6B2, BioLegend), anti-F4/80-FITC (clone BM8, eBioscience, San Diego, CA), anti–CD11b-PE (clone M1/70, BioLegend), anti–Ly-6G(Gr-1)-PECy7 (clone RB6-8C5, eBioscience), anti-CCR3-FITC (clone RB6-8C5, R&D System, Minneapolis, MN), and/or anti-Siglec-F-PE (clone E50-2440, BD Pharmingen, San Diego, CA) for 30 minutes at 4°C. Cells were analyzed using a BD LSRII flow cytometer and BD FACSDiva software. Data were analyzed using FlowJo v7.2.4 (Tree Star, Ashland, OR). Assayed proteins are listed in Table 1.

**Table 1:** Assayed proteins.

Protein (all *Mus musculus*)	Swiss-Prot Entry #
B220 (Ptprc)	Q05C79
CCR3	P51678
CD11b	P05555
CD3 epsilon chain	P22646
CD68	P31996
eotaxin	P48298
F4/80	Q61549
FoxP3	Q99JB6
G-CSF	P09920
GM-CSF	P01587
Gr-1 (Ly6G)	P35461
IFN-γ	P01580
IL-10	P18893
IL-12 beta subunit	P43432
IL-12 alpha subunit	P43431
IL-13	P20109
IL-17	Q62386
IL-1α	P01582
IL-1β	P10749
IL-2	P04351
IL-23	Q9EQ14
IL-3	P01586
IL-4	P07750
IL-5	P04401
IL-6	P08505
IP-10	P17515
KC	P12850
MCP-1	P10148
MCP-3	Q03366
MIP-1α	P10855
RANTES	P30882
Siglec-F	Q920G3
TGF-β	P04202
TNF-α	P06804
VEGF	Q00731

### Cytokine analysis

Rapidly-excised bladders were placed immediately on ice, minced in RNAlater solution (Qiagen), and stored at −80°C. For protein analysis, 50 mg of tissue was sonicated to homogeneity in 1 ml of ice-cold tissue extraction reagent (Biosource, San Diego, CA) supplemented with 1 mM phenylmethanesulfonyl fluoride. Clarified bladder extracts and serum samples were assayed using a mouse 26-plex cytokine kit (Affymetrix, Santa Clara, CA) according to the manufacturer's instructions. Samples were read using a Luminex 200 (Luminex, Austin, TX) with a lower cut off of 100 beads per sample (Human Immune Monitoring Core, Stanford University). Assayed proteins are listed in Table 1.

### RNA purification, cDNA synthesis, and real-time PCR

Regional lymph nodes were harvested and placed in RNAlater solution (Ambion, Austin, TX), and stored overnight at 4°C, then at −80°C for long-term storage. RNA was isolated and purified using RNAqueous -Micro kits (Ambion, Austin, TX) according to the manufacturer's instructions. The concentration of RNA was determined by Quant-iT RNA assay kit (Invitrogen, Eugene, OR) with the Qubit fluorimeter. The ribosomal RNA band integrity of each RNA sample was run on an Agilent Bioanalyzer using an RNA 6000 Nano Labchip. RNA samples with RNA Integrity Numbers (RIN) of 6 or higher were used for cDNA synthesis and real-time PCR arrays. cDNA synthesis was performed using the RT^2^ First Strand cDNA Kit (SABiosciences, Frederick, MD).

Real-time PCR was performed in the Mx3005p thermal cycler (Stratagene) using an RT^2^ custom PCR array (SABiosciences) with RT^2^ SYBR Green qPCR Master Mixes (SABiosciences). Cycle thresholds (Ct) were calculated for each reaction. Using the comparative Ct method relative gene expression was calculated as 2^(−ΔΔCt)^, where ΔCt = Ct (gene of interest) - ΔCt (normalizer = β-actin). ΔΔCt was calculated as ΔCt (egg-injected) - ΔCt (calibrator). Data are expressed as mean ± SD. P values are ΔCt of egg- versus control-injected mice. *, P<0.05; **, P<0.01; ***, P<0.005. Proteins corresponding to assayed genes are listed in Table 1.

### Voided spot on paper analysis

Voided spot on paper analysis was performed as previously described [76], [77]. In brief, mice underwent bladder wall injection with either eggs or control vehicle. One week later, mice were housed singly and acclimated for one hour in cages lined with filter paper laid underneath a wire floor bottom. Animals were given *ad libitum* access to food and water-soaked sponges placed on wire cage covers. After 8 hours, each piece of filter paper was photographed under ultraviolet light to localize voided urine spots. Total spots were counted for each mouse and the average number of voids was compared between the egg- and vehicle-injected mice using two-tailed T-tests. *, P<0.05; **, P<0.01; ***, P<0.005.

### Statistical analysis

Unpaired t tests with Welch's correction were used for comparisons between control- and egg-injected groups, and data were expressed as mean ± standard deviation. P<0.05 was considered statistically significant.

## Supporting Information

Figure S1 **Bladder wall injection induces hematuria and eosinophiluria.** At serial time points after bladder wall injection with eggs, cytospins were prepared from voided urine (n = 2–14/group), stained with Liu's stain, and cells identified by morphology. This demonstrated significant rates of eosinophiluria (A) and hematuria (B) in egg-injected mice. In contrast, neither eosinophils nor erythrocytes were present in voided urine from uninjected and control-injected mice (data not shown).
(TIF)

Figure S2 **Luminex analysis results of total bladder cytokine expression after bladder wall injection with** ***S. haematobium*** **eggs.**
(TIF)

Figure S3 **Luminex analysis results of serum cytokine expression after bladder wall injection with** ***S. haematobium*** **eggs.**
(TIF)

Video S1 **Micro-ultrasonography of a single representative animal day 1 post egg injection.** Video footage of micro-ultrasound probe scrolling along the z-axis of the lower abdomen of an egg-injected mouse demonstrates the presence of a bright, echogenic (dense) round granuloma impinging on the upper right side of the urine-filled (black), otherwise ovoid bladder lumen.
(WMV)
