# Supplementary figures and images for: A Novel Mouse Model of Schistosoma haematobium Egg-Induced Immunopathology
Source: PLoS Pathog. 2012 Mar 29;8(3):e1002605. doi: 10.1371/journal.ppat.1002605 (PMC3315496; doi:10.1371/journal.ppat.1002605)

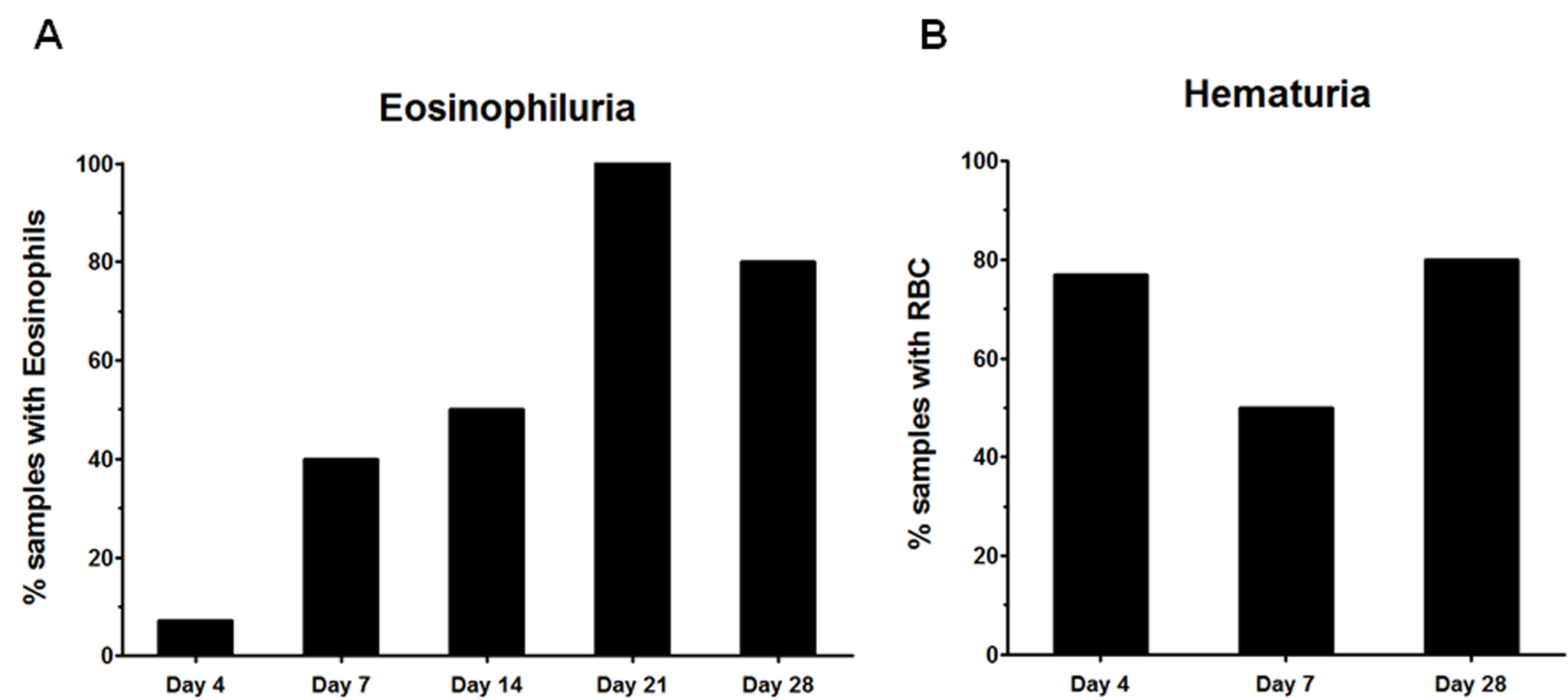

Supplement: Figure S1 — Bladder wall injection induces hematuria and eosinophiluria. At serial time points after bladder wall injection with eggs, cytospins were prepared from voided urine (n = 2–14/group), stained with Liu's stain, and cells identified by morphology. This demonstrated significant rates of eosinophiluria (A) and hematuria (B) in egg-injected mice. In contrast, neither eosinophils nor erythrocytes were present in voided urine from uninjected and control-injected mice (data not shown). (TIF) [file ppat.1002605.s001.tif]

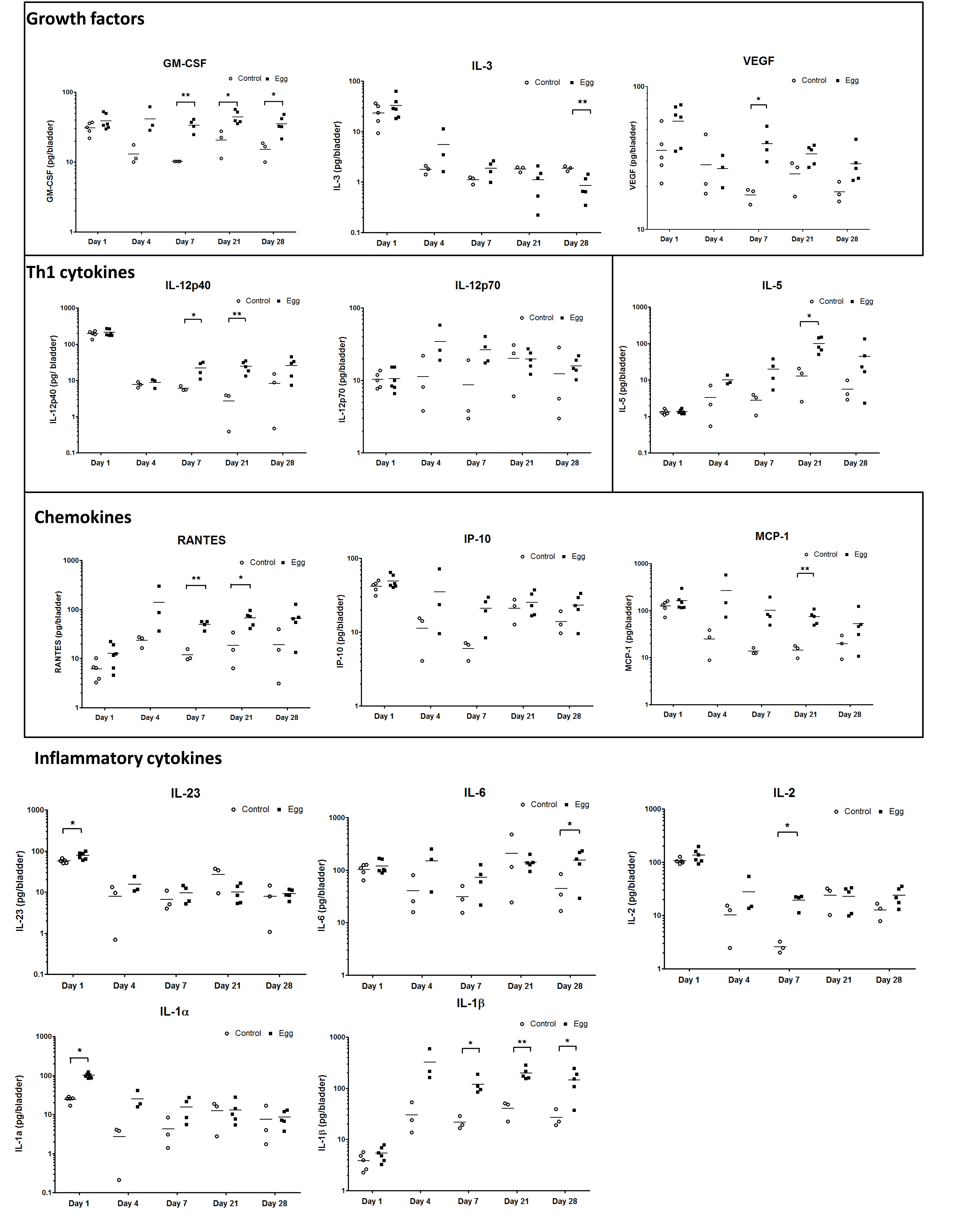

Supplement: Figure S2 — Luminex analysis results of total bladder cytokine expression after bladder wall injection with S. haematobium eggs. (TIF) [file ppat.1002605.s002.tif]

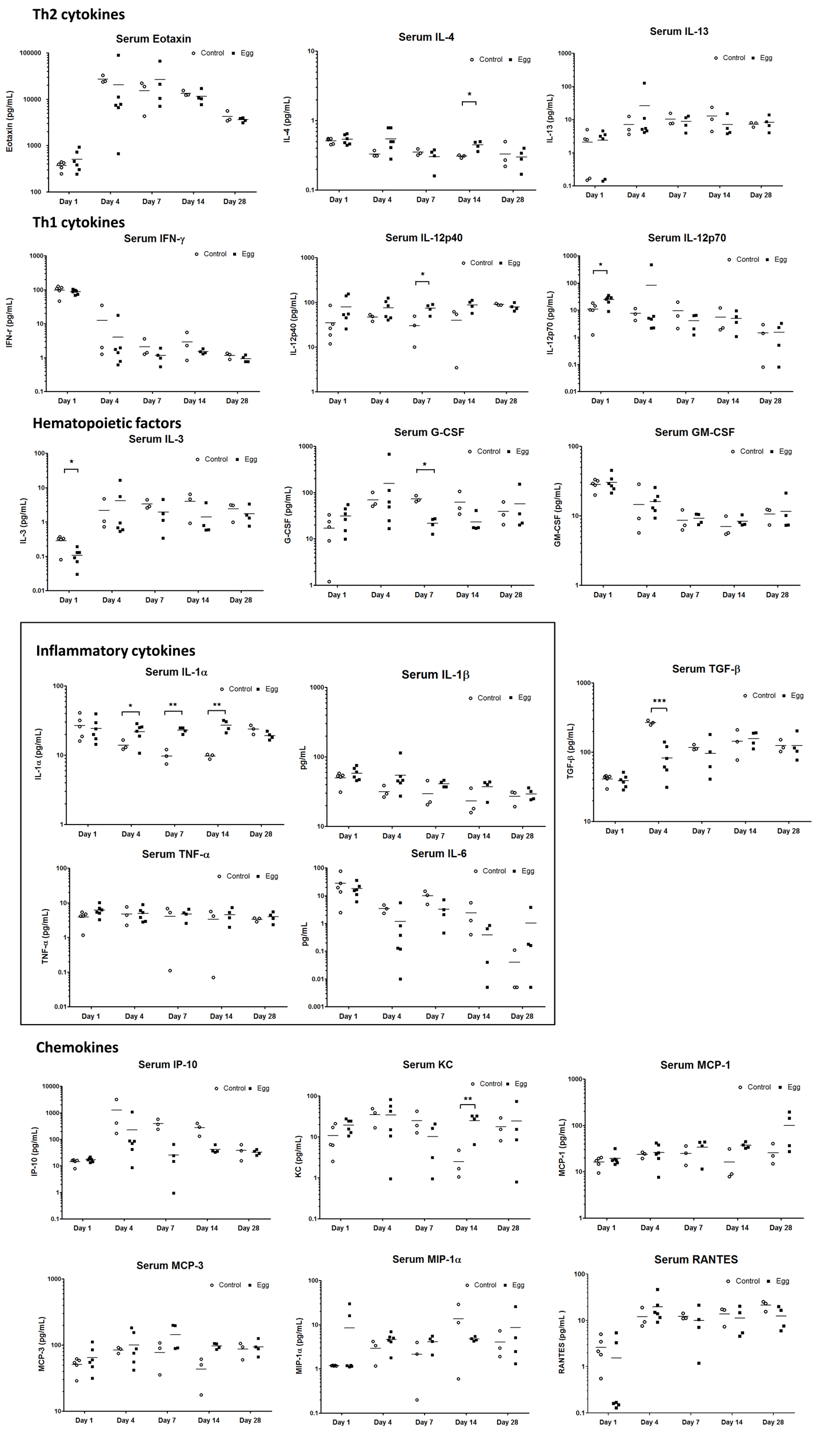

Supplement: Figure S3 — Luminex analysis results of serum cytokine expression after bladder wall injection with S. haematobium eggs. (TIF) [file ppat.1002605.s003.tif]
